# Gut Virome: Role and Distribution in Health and Gastrointestinal Diseases

**DOI:** 10.3389/fcimb.2022.836706

**Published:** 2022-03-10

**Authors:** Laurie Spencer, Babatunde Olawuni, Pallavi Singh

**Affiliations:** Department of Biological Sciences, Northern Illinois University, DeKalb, IL, United States

**Keywords:** virome, bacteriophage, eukaryotic virus, gastro-intestinal disease, phage therapy

## Abstract

The study of the intestinal microbiome is an evolving field of research that includes comprehensive analysis of the vast array of microbes – bacterial, archaeal, fungal, and viral. Various gastrointestinal (GI) diseases, such as Crohn’s disease and ulcerative colitis, have been associated with instability of the gut microbiota. Many studies have focused on importance of bacterial communities with relation to health and disease in humans. The role of viruses, specifically bacteriophages, have recently begin to emerge and have profound impact on the host. Here, we comprehensively review the importance of viruses in GI diseases and summarize their influence in the complex intestinal environment, including their biochemical and genetic activities. We also discuss the distribution of the gut virome as it relates with treatment and immunological advantages. In conclusion, we suggest the need for further studies on this critical component of the intestinal microbiome to decipher the role of the gut virome in human health and disease.

**Graphical Abstract:**
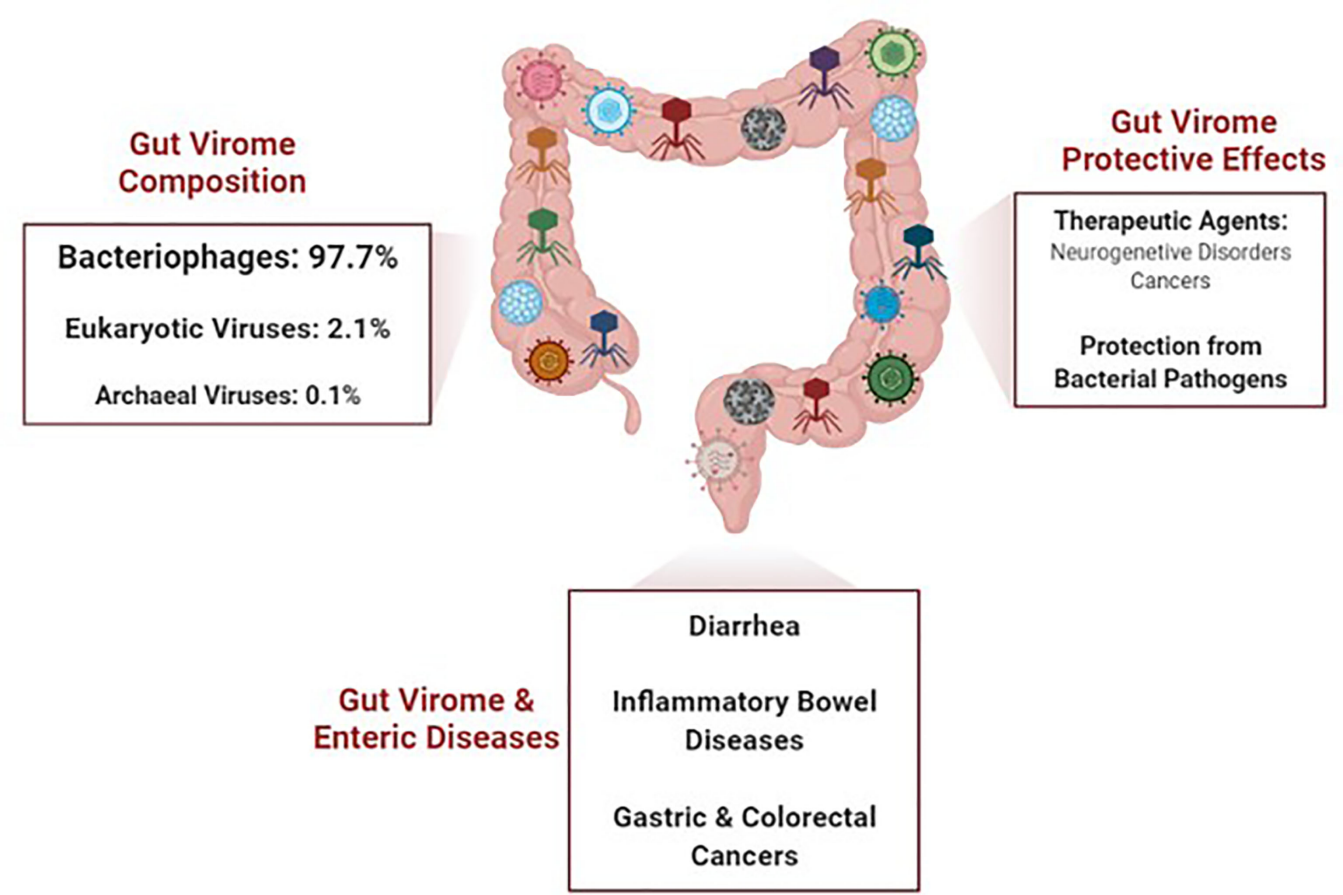


## Introduction

The human gut virome consists of the total population of viruses and their genomes that are found throughout the gastrointestinal tract. It is estimated that the human intestines harbor about 35 – 2800 active bacterial viruses in 1g of feces (Kim et al., 2011; Manrique et al., 2016). The intestinal virome includes both bacteriophages (hereafter referred to as phages) and eukaryotic viruses. Maintaining homeostasis in the complex intestinal microbial environment plays a significant role in improving the health status of the host which could otherwise contribute to the development of disease conditions (Weinbauer, 2004). Major clinical conditions such as diabetes and Crohn’s diseases have been associated with the disruption in the composition of commensal flora of the gut (dysbiosis) (Frank et al., 2007; Larsen et al., 2010; Perez-Brocal et al., 2013). Dysbiosis leads to dynamic changes in the gut community as well as phage activities (Flores et al., 2011; Ogilvie and Jones, 2015). Bacterial populations in the gut are controlled by various factors ranging from the host dietary content to the immune system and the predatory effects of phages. For example, 10-80% of total bacterial death in nature can be attributed to phage attack, hence signifying their roles in microbial community (Weinbauer and Rassoulzadegan, 2004). Further, phages are involved in the lateral transfer of genes between bacteria and serve as a determinant for genetic variability (Canchaya et al., 2003). Several recent studies have reported the role the virome, particularly phages, plays in the development of certain clinical conditions, including bowel disorders and cancer (Frank et al., 2007; Tong et al., 2013; Brooks and Watson, 2015). In this review, we provide a comprehensive compilation of the role that the virome plays in GI health and diseases.

## The Virome Development

Microbiome (virome inclusive) composition varies with age, dietary intake, host immunological status, drug intake, and environmental factors. Major bacterial phyla that dominate the gut at the early stage of infancy include, *Firmicutes*, *Bacteroidetes*, *Actinobacteria*, *Verrucomicrobia*, *Proteobacteria* and *Fusobacteria*. Phage-bacteria relationship at this stage is inversely related where an increase in phage density is observed with low bacterial population and vice-versa. This relationship indicates that the prey (bacteria) distribution influences the predator’s (phage’s) diversities (reversed predator-prey dynamics) (Lim et al., 2015). The Global Virome Database indicates that 97.7% of the human gut virome are phages, 2.1% are eukaryotic viruses, and 0.1% are archaeal viruses; 88% of these phages have yet to be classified by the International Committee on Taxonomy of Viruses (Gregory et al., 2020).

**Figure 1:**
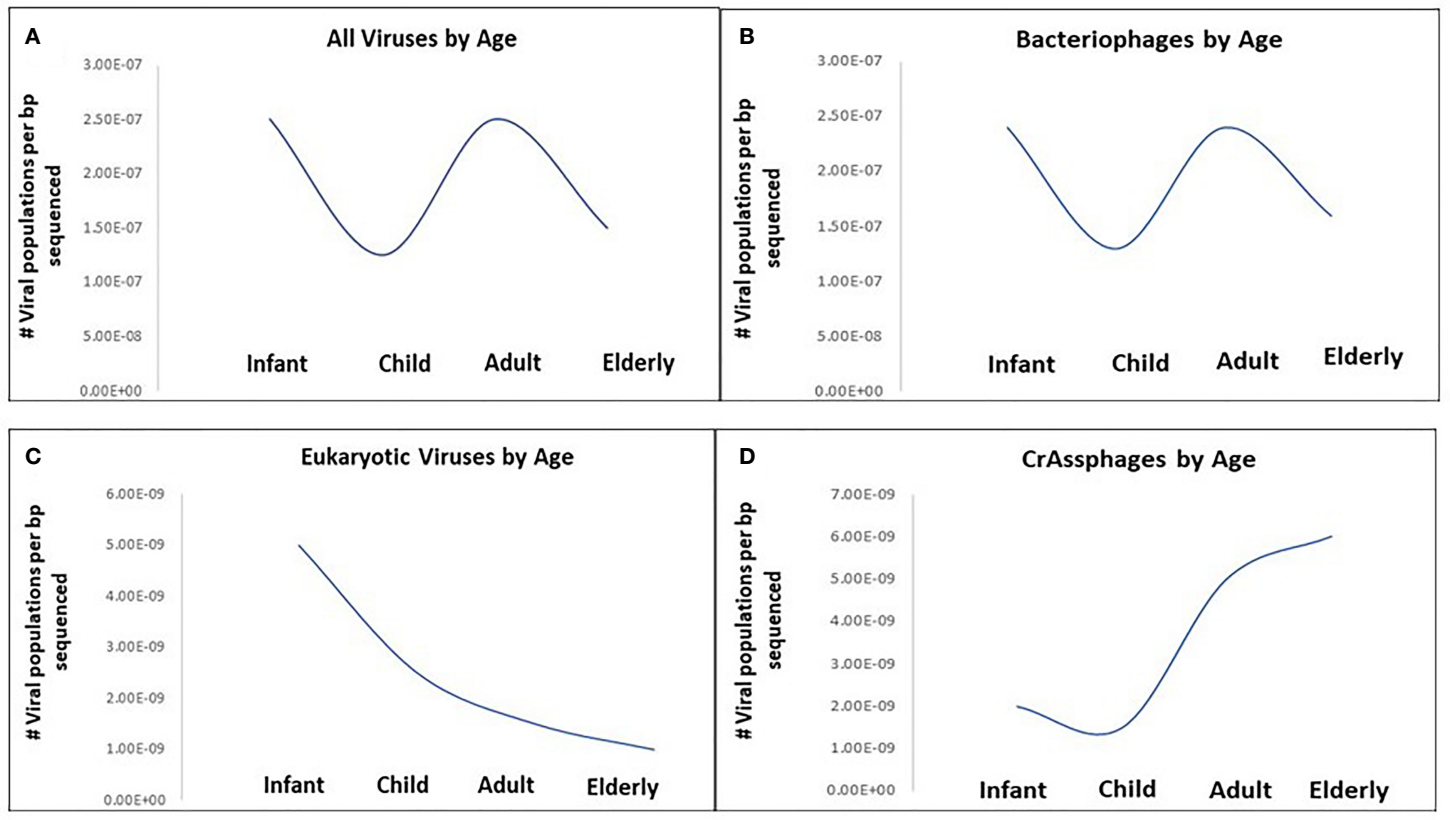
Viral richness changes with age in healthy humans across four age categories: Infant (0-3 years), Child (3-18 years), Adult (18-65 years) and Elderly (65+ years). **(A)** All viruses by age, **(B)** bacteriophages by age, **(C)** eukaryotic viruses by age, **(D)** CrAssphages by age. Viral richness quantified as the median number of viral populations per base-pair sequenced for each age category based on results from Gregory et al., 2020.

The dynamism of human virome progresses from childhood to adulthood. The colonization of the human intestine by microbes starts right after delivery with usually very low populations of microorganisms consisting mainly of bacteria (Jimenez et al., 2008). Gregory et al. determined that infants (0-3 years) and adults (18-65 years) show higher viral richness, with decreases for children (3-18 years) and the elderly (65+ years). Bacteriophage richness followed this trend whereas eukaryotic virus richness was high in infants and steadily decreased throughout life (**Figure 1**) (Gregory et al., 2020).

Gut virome in neonates initially is comprised of phages which infect the pioneer bacteria, followed by eukaryotic virus diversification associated with environmental exposures, particularly breastmilk (Liang et al., 2020). The immediate environment contributes to the diversity of the gut phage among the neonates (Huurre et al., 2008; Lim et al., 2015). Other studies in children (ages 0 to 3 years) have reported the predominance of DNA phages from the *Caudiovirales* order, mostly comprising of *Myoviridae*, *Siphoviridae* and *Podoviridae* family (Reyes et al., 2015). High phage abundance of *Caudiovirales*, and to a lesser extent of *Microviridae*, in this age group is attributed to the early colonization of the gut by *Bacteroides*, *Proteobacteria* and *Actinobacteria* bacteria (Reyes et al., 2010; Lim et al., 2015). Less abundant DNA eukaryotic viruses in the *Anelloviridae* and *Herpesviridae* families have also been detected in infant stool (Breitbart et al., 2008). The single-stranded (ss) DNA viruses of the *Anelloviridae* family (particularly Torque Teno Virus species) has been shown to be the most abundant in the few months after birth (Lim et al., 2015). Prevalence of *Anelloviridae* is directly associated with host immunosuppression, and pediatric febrile illness (McElvania TeKippe et al., 2012; De Vlaminck et al., 2013). This early onset of DNA viruses is attributed to immature host immune system, and its subsequent reduced load correlates to development of a fully competent immune response (Fulci et al., 2021).

Recently, highly divergent phages have been identified among healthy children, for instance, *CrAssphage* which are associated with *Bacteroides* (Dutilh et al., 2014). *CrAssphages* richness showed an overall upward trend with age; are abundant and persistent in the human gut virome, and functions to maintain a stable population of both resistant and sensitive bacterial hosts (**Figure 1**) (Gregory et al., 2020; Shkoporov et al., 2021). Metagenomic analysis of the eukaryotic viruses revealed that the diversities of the viral population are influenced by the geographical location and the health status of the host. A study on Amazonian children demonstrated the abundance of *Picornaviriridae* and *Caliciviridae* families (Siqueira et al., 2018). Other eukaryotic viruses recovered in the healthy human gut include *Circoviridae, Anelloviridae*, *Reoviridae* and *Astroviridae* (Altan et al., 2018). Although Breibart et al. claimed that vertical transmission of the ssDNA eukaryotic viruses, particularly *Anelloviridae*, occur during vaginal delivery from mother to child; factors that promote their increased abundance following first few months after delivery are unknown (Breitbart et al., 2008). Further investigations are required to identify the mechanism involved in this colonization. Furthermore, the role of internal factors such as peristalsis, immunity, drug regimen etc., on virome composition need further assessment due to their importance in gut diversity (Mackie et al., 1999).

Progressively, the microbial community in the infant gut undergoes a series of developmental shifts following the dietary change from liquid to solid (Sharon et al., 2013). These developmental shifts lead to the emergence of a balanced microbiome that is stable compositionally and functionally (Mackie et al., 1999; Minot et al., 2013). At this stage, the most commonly identified bacteria in healthy adult subjects are *Bacteroidetes* and *Firmicutes* (Mackie et al., 1999) and their dominance subsequently affects the virome composition (Minot et al., 2013). The heterogeneity in gut virome is also attributed to phages with single-stranded DNA, specifically *Microviridae* due to high mutations rates (Minot et al., 2013). Manrique et al. analyzed DNA phage in healthy adult stool and reported that the gut composition is made up of *Podoviridae, Siphoviridae, Myoviridae*, and *Microviridae* families. Additionally, Zhang et al., reported the abundance of *Virgaviridae* viruses of plant origin as the common eukaryotic RNA virus in the gut of the healthy adults suggesting dietary influence (Zhang et al., 2006). Pepper mild mottle virus (PPMV) was predominantly detected among these families whereas less common RNA viruses identified included maize chlorotic mottle virus (*Tombusviridae*), oat chlorotic stunt virus (*Tombusviridae*), grapevine asteroid mosaic virus (*Tymoviridae*), and panicum mosaic virus (*Tombusviridae*) (Zhang et al., 2006). The stability of the virome in the adult could contribute to the positive health status of the human host, however, a disturbance of the viral community may result in gut microbiome dysbiosis (Flores et al., 2011).

## The Virome and the Human Host Interaction

The human body serves as a reservoir of many microorganisms including viral communities (Weinbauer and Rassoulzadegan, 2004; Pride et al., 2012; Tremaroli and Backhed, 2012). Metagenomic shotgun sequencing provides a platform to investigate the genetic potentials of indigenous viral community and aid in the identification of distinct new phages (Dinsdale et al., 2008; Perez-Brocal et al., 2013; Bibby, 2014).The most common human gut phages are grouped into three main families based on their tail structure: a complex extensive-tailed *Myoviridae*, long non-extensive tailed *Siphoviridae*, and short non-extensive tailed *Podoviridae* (Veesler and Cambillau, 2011). These are double-stranded DNA phages that belong to *Caudovirales* order and account for approximately 95% of the bacteriophages (Maniloff and Ackermann, 1998). Single-stranded DNA phage families including *Microviridae* and *Inoviridae* have also been described (Kim et al., 2011; Szekely and Breitbart, 2016; Creasy et al., 2018). However, metagenomic studies that focus on the RNA phages in the human gut are relatively few (Zhang et al., 2006; Manrique et al., 2017). Additional studies on the human gut virome have revealed that diverse genotypes of phages stably reside and play a distinct role in maintaining the host health (Minot et al., 2012; Abeles and Pride, 2014). Heterogeneity in the viral composition in other body sites such as the phages detected in skin surfaces, have also been described (Oh et al., 2016). Human microbiome compositions, including virome, are reportedly influenced by various factors such as gender, age, environmental reservoir, diet, human-to-human contact, immune status, and the health of the hosts (Breitbart et al., 2008; Arthur et al., 2009; Azad et al., 2013; Abeles et al., 2014; Hodyra-Stefaniak et al., 2015; Creasy et al., 2018). Host sex was found to be associated with changes in composition and diversity of the viral community, particularly in the oral virome, likely due to hormonal effects on the bacterial community (Abeles and Pride, 2014). Further, a comparative study involving the analysis of lactococcal virus in infant and adult stool samples shows an increased abundance of *Myoviridae* family probably due to change in diet (Deveau et al., 2006).

## The Phage Impacts on Enteric Bacteria Virulence

Phages play a major role in bacterial evolution and exhibit viral tropism, which is influenced by bacterial rigidity, phage competence and other environmental conditions (Weinbauer, 2004; Rohwer et al., 2009; Lee et al., 2016). Phage infection may either be deleterious or beneficial to the bacteria as a result of lateral gene transfer within the bacteria community (Canchaya et al., 2003). Phages may encode toxin genes which are important in the pathogenicity of many bacteria (Gyles, 2007). The virulence effect produced by toxin-inducing prophage is best described in the role they play in Shiga toxin-producing *Escherichia coli* (STEC) virulence. STEC is a clinically significant foodborne pathogen which produces complications like hemolytic uremic syndrome and may be fatal in severe cases, due to the conferred effects of lambdoid phage encoded *shiga-toxin* (*stx*) genes (Riley et al., 1983; Gyles, 2007). The use of antibiotics (e.g., norfloxacin) may induce lytic cycle of stx-prophage, which can ultimately lead to fatal morbidity in the infected individuals (Zhang et al., 2000). A similar toxin-producing mechanism is observed in bacteria such as *Staphylococcus aureus* and *Vibrio cholera* with toxic shock syndrome toxin (tst) and accessory cholera exotoxin (*ace*), respectively (Trucksis et al., 1993; Ruzin et al., 2001). Phages can also encode for virulence factors within host bacterial cells, for instance, the temperate phage, Sopφ, encodes for *sopE* effector protein production which facilitates the entry of *Salmonella* spp. into the host intestinal epithelium (Wood et al., 1996; Hardt et al., 1998). Phages may also increase the pathogenicity of bacteria directly through the virion particle structures (e.g., hyaluronidase tightly bound to the phage) (Benchetrit et al., 1977), or by replication and transcription of phage-encoded genes as in the case of diphtheria toxin produced by *Corynebacterium diphtheriae* (Wagner and Waldor, 2002). Faruque et al. reported a change of a non-virulent strain of *Vibrio cholera* to a virulent type following *CTXPhi* (filamentous) phage induction (Faruque et al., 1998). Apart from horizontal transfer of virulence genes to non-pathogenic strains, several studies have demonstrated that virulence genes can also be transduced within bacterial community members. Examples include the transfer of *stx* genes among the *Enterobacteriacae* family members and an opportunistic bacteria, *Acinetobacter haemolyticus, via* transduction (James et al., 2001; Gamage et al., 2004; Grotiuz et al., 2006; Gorski et al., 2016). Both these bacteria cause bloody diarrhea (James et al., 2001; Grotiuz et al., 2006).

Phages can contribute to pathogenicity in bacteria by encoding genes which serve as disease factors. Phages of *Staphylococcus aureus*, *sakϕ*C and *sak*42D, encode for the immune regulator staphylokinase (*sak*) which can counteract host immune responses by neutralizing antimicrobial peptides (Nguyen and Vogel, 2016) and cause host tissue death (van Wamel et al., 2006). Other phage-encoded substances, such as complement inhibitors (SCIN), protein inhibitors (CHIP) including superantigens are known to be associated with lethal outcome of *S. aureus* infection (de Jong et al., 2018). Phages have been reported to promote establishment of diseases by mediating biofilm formation, thus increasing adhesion and resistance to antibiotics and desiccation, of bacteria like *Pseudomonas aeruginosa* and *E. coli* in humans with cystic fibrosis (Secor et al., 2015). In mice, Waldor and Mekalanos demonstrated that filamentous phage (*CTXF*) is involved in the entry of *V. cholera* into the host’s epithelial tissue through the binding of toxin co-regulated pili (TCP) receptor (Waldor and Mekalanos, 1996).

Another mechanism by which phages increase enteric bacterial pathogenicity is through the transfer of genes encoding antibiotics resistance or genes that evade the immune system (Colomer-Lluch et al., 2011). For instance, Marta et al., reported that β-lactamase genes, *blaTEM* and *blaCTX-M9* in the *S. aureus* phage can be laterally transferred into environmental isolates conferring ampicillin resistance to new *S. aureus* strains. Temperate phages have also been implicated as major carriers of transducing multi antibiotic-resistant genes among *Salmonella* Typhimurium strains (Colomer-Lluch et al., 2011). A study on the role of virome in bacterial adaptation, after antibiotic-induced stress in animal models revealed that phage genes undergo robust enrichment following antibiotic treatment. Such gene enrichment could promote the production of resistant gene against the administered drug in phages and their subsequent transfer to the bacterial community, thereby acting as a reservoir for resistant strains (Modi et al., 2013).

## The Virome and Enteric Diseases

The eukaryotic viruses that are involved in enteric infections are known as enteric viruses. In infected subjects, about 10^6^ – 10^8^ VLPs of eukaryotic viruses are usually shed in each gram of stool (Farthing, 1989). The most frequently detected eukaryotic viruses in acute gastroenteritis are *Reoviridae* (rotavirus), *Picornaviridae* (enterovirus, echovirus etc.), *Adenoviridae* (adenovirus) and *Caliciviridae* (norovirus) (Wilhelmi et al., 2003; Finkbeiner et al., 2008a). Phages also play a significant role in the study of intestinal diseases in several ways by: (1) acting as a major determinant of virulence in enteric infections (2) associating with dysbiosis in gut during mild and chronic inflammatory bowel disease (IBD) (3) being employed as a therapeutic regimen for many human infections including intestinal disease. The human GI tract harbors abundant of diverse microbial populations that are involved in shaping human health. It is estimated that human fecal samples carry up to 10^5^ phage per gram dry feces that specifically attack different strains of *E. coli, Salmonella* spp. and *B. fragilis* bacteria (Havelaar et al., 1990; Calci et al., 1998). The diversity of the gut virome is evolving as a major subject for host health, and its disruption has been shown to be related with disease conditions in both human and animal models (Turnbaugh et al., 2006; Tong et al., 2013; Kim and Bae, 2018). Apart from promoting virulence of the pathogens, the distribution of phages in the gut are also attributed to different disease progression, immune system functions and gut homeostasis (Gorski et al., 2016; Zhao et al., 2017; Metzger et al., 2018). The role of the virome has been implicated in intestinal conditions and even related diseases such as Crohn’s disease (Dejea et al., 2014; Gorski et al., 2016; Nakatsu et al., 2018) and ulcerative colitis (Frank et al., 2007; Brooks and Watson, 2015; Sheehan et al., 2015; Gorski et al., 2016). In this section below, we elaborate on the role of gut virome in enteric infections (**Table 1**).

**Table 1:** Increased viral loads associated with GI diseases (* indicates novel virus).

	Bacteriophage	Eukaryotic Virus	References
**Diarrhea**	N/A	Anellovirus Adenovirus Calicivirus Astrovirus Rotavirus Picobirnavirus* Norovirus Torque Tenovirus Enterovirus Dependovirus Sapovirus Bufavirus* Bocavirus*	(Finkbeiner et al., 2008; van Leeuwen et al., 2010; Phan et al., 2012; Holtz et al., 2014)
**Ulcerative Colitis**	Caudiovirales	Virgaviridae Anelloviridae Circoviridae Picobirnaviridae	(Norman et al., 2015; Conceicao- Neto et al., 2018)
**Crohn's Disease**	Caudiovirales Siphoviridae Myoviridae Podoviridae	N/A	(Lepage et al., 2008; Norman et al., 2015)
**Gastric & Colorectal Cancer**	Inovirus Tunalikevirus	Herpesviridae Cytomegalovirus Epstein-Bar virus Human papilloma virus Polyomavirus Orthobunyavirus	(Moritani et al., 1996; Rollison, 2010; Damin et al., 2013; Abedon et al., 2017; Nakatsu et al., 2018; Emlet et al., 2020)

N/A stands for not applicable.

### Diarrhea

Globally, acute diarrhea is one of the health conditions with highest mortality rate, particularly among children, and viruses are identified to be one of the causative agents. In about 40% of reported diarrheal disease cases etiological agents are unidentified, however the use of metagenomics sequencing technique has enabled detection of novel viruses that may be responsible for acute infections (Finkbeiner et al., 2008b). Finkbeiner et al, studied the viral community of the fecal samples from patients with acute diarrhea and detected phages as well as common eukaryotic viruses such as, anellovirus, adenovirus, calicivirus, astrovirus and rotavirus, which belong to *Anelloviridae*, *Adenoviridae, Caliciviridae: Astroviridae*, and *Reoviridae* families respectively. Putative novel DNA viruses identified here, shared homology with picobirnavirus, norovirus, Torque Teno virus, and *enterovirus* genus, while the detected RNA virus was related to *Nodaviridae* family (Finkbeiner et al., 2008b). In addition to these, van Leeuwen et al., reported the identification of *Picornarviridae, Retroviridae*, and a novel picobirnavirus variant with distinct phylogenetic relatedness in patients with diarrhea, indicating the involvement of new emerging viruses (van Leeuwen et al., 2010). The distribution of viruses in diarrhea have been attributed to factors including population, age, etiological agent and other environmental conditions. For example, a study at two geographical locations in Australia in pediatric patients, exhibited abundance of *Adenoviridae* and *Picornaviridae* families with varying proportion (Holtz et al., 2014). In another study, Phan et al., observed that anelloviruses and dependovirus were most prevalent in West African children with acute diarrhea closely followed by sapoviruses, enteroviruses and bocaviruses (Phan et al., 2012). It is noteworthy that both anelloviruses and dependoviruses are themselves not pathogenic but have been detected by several studies and are presumably markers of infection. The mechanisms by which these viruses induce diarrhea are not well understood, however, rotavirus and adenovirus infections within enterocytes of the small intestine result in atrophy of the villi and crypt cell hyperplasia leading to fluid malabsorption (Wilhelmi et al., 2003). Novel viruses such as bufavirus and bocavirus share homology with *Parvoviridae* family have also been recovered in the feces of patient with diarrhea. The detection of these novel viruses depict diversity in *Parvoviruses*, however their possible role in this clinical condition is unknown (Arthur et al., 2009; Phan et al., 2012).

### Inflammatory Bowel Disease

IBD inclusive of ulcerative colitis (UC) and Crohn’s disease (CD) is a chronic clinical condition that causes recurring inflammation of the intestine (Abraham and Cho, 2009). A common feature of CD includes a rough patch-like appearance of the inflamed tissues of the intestine. This inflammation can cause perforation of the intestinal wall and have a resultant effect on vital organs such as kidneys and uterus. Development of UC is localized in the colon where dysbiosis occurs (Nagalingam and Lynch, 2012). Intestinal microbiome and virome are crucial for human health and have been implicated to be significant factors in the IBD progression (Lepage et al., 2008). Elevation of *Caudiovirales* phages was found in IBD patients, particularly with CD, accompanied by a decrease in bacterial diversity, thereby demonstrating the possible influence of phages in these conditions (Lepage et al., 2008; Sheehan et al., 2015). Comparative analysis of the Viral-like particles (VLP) from the biopsy of CD patients exhibited an increase in *Siphoviridae*, *Myoviridae* and *Podoviridae* in patients as compared to the healthy control groups. Further, metagenomics studies revealed mild disparity in the diversity and richness in the viral composition of *Caudiovirales* taxa between UC and CD patients (Norman et al., 2015). This suggests that the variation in the virome may serve as a biomarker for classifying these clinical conditions. Normal et al., reported that a converse relationship exists between phage richness and bacteria diversity in CD where the rate of growth of *Bacteroidaceae* is greatly reduced in presence of *Caudiovirales* (i.e., negative phage-bacteria correlation). However, *Caudiovirales* population in UC showed positive correlation with the growth of *Enterobacteriacae*, *Pastaurellacaea* and *Prevotellaceae* (Brooks and Watson, 2015; Gorski et al., 2016).

Eukaryotic viruses, particularly *Anelloviridae*, are also reported to be higher in IBD patients as compared to the healthy individuals, although their role in IBD condition is not known (Norman et al., 2015). Based on the observed viral richness, diversity between UC patients undergoing fecal microbial transplant (FMT) and healthy control group, Conceicao et al., suggested that the eukaryotic viral richness could serve as a potential biomarker in diagnosis of UC (Norman et al., 2015; Conceicao-Neto et al., 2018). The four dominant viral families reported in this study include, *Virgaviridae, Anelloviridae, Circoviridae*, and *Picobirnaviridae (* Conceicao-Neto et al., 2018 *)*. While these studies have increased our knowledge about the distribution and possible involvement of the viruses in IBD, future investigations are warranted to discuss roles that diet, age, immunity plays in shaping IBD.

### Gastric and Colorectal Cancer

In US alone, death due to cancer accounts for the second largest cause with GI cancer being associated with high morbidity and mortality rate (Siegel et al., 2015). Studies on the gut bacterial distribution and pathogenesis have shown that dysbiosis leads to development of colorectal cancer (CRC) (Baxter et al., 2016; Yu et al., 2017). Additionally, 15-20% of cancer incidence worldwide is associated with oncogenic virus infections (Chen et al., 2019). Oncogenic DNA viruses can cause cancer by interfering with cellular division or DNA repair mechanisms, while RNA viruses do so through the production of reactive oxygen species or chronic inflammation (McLaughlin-Drubin and Munger, 2008). Knowledge of the virome therefore provides useful information in the CRC screening particularly in the early stages (Zackular et al., 2014; Zeller et al., 2014; Yu et al., 2017). The virome has been associated in the development of two major GI cancer: gastric cancer and CRC (Moritani et al., 1996; Bull and Plummer, 2014; Zackular et al., 2014). The eukaryotic virus, Epstein-Bar virus (Herpesvirus), is a known etiological agent of gastric cancer (Moritani et al., 1996). Nakatsu et al. revealed that phage richness (due to the significant detection of distinctive members of *Inovirus* and *Tunalikevirus*) is common in CRC patients compared to the control group (Nakatsu et al., 2018). *Inovirus* species are tiny filamentous phages that are associated with regulating bacteria exopolysaccharide matrix synthesis, a precursor for biofilm formation, an underlying factor in colorectal tumor development (Dejea et al., 2014; Johnson et al., 2015; Secor et al., 2015). Enteric phages that target *Bacteroides fragilis*, *Fusobacterium nucleatum* and *Escherichia coli* have been associated with CRC development; the speculative mechanism of oncogenesis being phage ability to directly transfer into colonic epithelial cells as well as phage encoding for virulence genes, particularly genes regulating biofilm production (Emlet et al., 2020).

*Orthobunyavirus*, a eukaryotic virus,was uniquely abundant in CRC patients and may be used as a marker for CRC diagnosis (Nakatsu et al., 2018). *Herpesviridae*, eukaryote virus family, were more predominant in CRC, particularly *Cytomegalovirus* spp., which are usually implicated as an etiological agent (Dejea et al., 2014). Human papillomavirus infections were found to be associated with increased risk of CRC (Damin et al., 2013) by integrating into the host genome, but the mechanism for induction of cancer is not well understood (Emlet et al., 2020). Polyomaviruses can be oncogenic due to their encoding of T-antigen which can inactivate the p53 and pRB tumor suppressor proteins (Rollison, 2010) leading to unregulated tumor growth.

## Protective Effect of the Virome

Health conditions such as cancer and neurodegenerative disorders have been treated using phage therapy with promising outcomes. For example, a study exhibited the binding potential of M13 to both β-amyloid and α-synuclein proteins, in the brain of non-human primates (*Macca mulatta*) suggesting phage therapeutic use in neurodegenerative disorders (Alzheimer’s and Parkinson’s diseases) (Ksendzovsky et al., 2012). In the treatment of cancer cells in mice tumor models, delivery phages have been engineered to transfer anticancer protein into the cancer cells either for malignant tumor atrophy or cancer gene therapy, thereby facilitating the cell death. In addition, phage can play a role in bolstering the immune system by preventing the reactive oxygen species (ROS) synthesis in endotoxin induced oxidative stress (Dabrowska et al., 2004). The use of phages as therapeutic agents has been extensively reviewed (Abedon et al., 2017). In this section, we focus on the role of phages in ameliorating GI tract (GIT) disorders and associated possible drawbacks with their usage.

### Treatment of Intestinal Diseases

Phages have been used to treat infectious diseases of the intestines and skin in Eastern European and former Soviet Union countries since the early 1900s (Morozova et al., 2018). The long history of phage research spans many years in various countries; phages have been used with notable success to treat bacterial dysentery in France (1919), cholera in India (1927), acute colitis in Georgia (1936), and bacterial dysentery, acute colitis and salmonellosis in Russia (1968-1993) (El-Shibiny and El-Sahhar, 2017). Some prospective lysogenic phages have been used in formulating therapeutic cocktails because of their effectiveness in deleting virulence genes and adding short chain genes necessary for fatty acid metabolism in certain pathogenic bacteria (Regeimbal et al., 2016). Protective phage action was demonstrated in the evasion of *Salmonella* colonization in anaerobically cultivated tissue culture and had minimal or no effect on non-target bacteria (Hu et al., 2018). Phage treatment can be adapted in managing enteric diseases caused by bacterial pathogens like *V. cholera*e (Summers, 2001), *Salmonella* spp. (Goode et al., 2003); *S. aureus* (Mann, 2008; Oh et al., 2016), *Clostridioides difficile; Listeria monocytogenes* (Carlton et al., 2005; Mai et al., 2010), *Campylobacter jejuni* (Goode et al., 2003; Hwang et al., 2009; Gorski et al., 2016) and *E. coli* (Sarker et al., 2016). Therapeutic use of phage products directly in humans is not approved in most countries, including United States. However, commercially approved phage-based formulations are produced in a number of countries which can prevent food-borne intestinal infections. These phage products control bacterial pathogen contamination to increase food safety: *E. coli* O157:H7 (EcoShield^TM^ by Intralytix, USA), *Listeria monocytogenes* (ListShieldTM by Intralytix, USA, and LISTEXTM P100 by EBI, Netherlands) and *Salmonella* spp., from swine and poultry animals (BioTector by CheilJedang Corporation, Korea) (Monk et al., 2010; Endersen et al., 2014; Nannapaneni and Soni, 2015). Commercially available *E. coli*-targeting phages (PreforPro®) has been given experimentally to humans reporting gastrointestinal distress, showing no global disruption of the microbiota and positive outcomes, specifically, increased butyrate-producing *Eubacterium* and decreased bacteria closely related *Clostridium perfringens* (Febvre et al., 2019). The CRISPR-Cas system of *E. coli* is being researched with the goal of reducing drug-resistant pathogens using phages to insert the CRISPR-Cas program targeting bacterial resistance genes (Yosef et al., 2015).

On the other hand, the use of phage therapy has received many critical reviews which has limited its use both commercially and globally (Sulakvelidze et al., 2005; Kutter, 2008). Safety of the phage (Meader et al., 2013; Abedon et al., 2017), pharmacodynamics impact of phages (Carey-Smith et al., 2006), and host immunological compatibility with the formulated phages are major concerns (Hodyra-Stefaniak et al., 2015). Since, phage can infect many bacteria, the challenge of stringent specificity for target cells is hindering the use of phage regimen (Garcia et al., 2009; Gorski et al., 2016). Another setback in the use of phage is the rapid evolvement of mutant strains that are resistant to the phage treatment (Garcia et al., 2009; Kocharunchitt et al., 2009). Therefore, more studies are needed in this area before the phage therapy can be globally acceptable.

### The Virome Induced Immunogenicity

The importance of viruses and phages in conferring immunity are well documented in studies involving mouse models as well as human subjects. Experimental mouse studies have suggested that enteric viruses can provide beneficial effects. For example, murine norovirus (MNV) infection was shown to reverse bacterial dysbiosis-induced GIT disease by repairing Paneth cell and crypt-villi functionality in the small intestines by upregulating the production of IFNγ and IgA (Kernbauer et al., 2014). Other investigators observed mice with latent murine gammaherpesvirus 68 or murine cytomegalovirus (mCMV) infections were protected against infection by the bacterial pathogens, *Yersinia pestis* and *Listeria monocytogenes via* persistent immune stimulation from macrophage activity and antiviral IFNγ (Barton et al., 2007). In studies involving phage effects, patients with *C. difficile* infections were treated with fecal filtrate showed clinical improvement and significant changes in their intestinal phage community resembling the fecal donor (Ott et al., 2017). In another study, patients with various *Staphylococcus* spp. infections underwent experimental phage treatment with a staphylococcal phage cocktail (MS1) resulting in increased antibody stimulation (mainly IgG and IgM) (Żaczek et al., 2016). Mucosal surfaces can be entry points for invading pathogens; increased concentrations of mucus-adherent phages have been shown to provide host immune defense against bacterial infections (Barr et al., 2013; Barr et al., 2015). Filamentous phages have experimentally shown much potential for the development of vaccines and can be engineered as therapeutic agents for managing bacterial infections and chronic diseases, such as cancer, Alzheimer’s disease, and Parkinson’s disease. These phage based vaccines have capacity for displaying various surface antigens, like bacterial LPS encoded on phage coat, to provoke various immune responses including innate immunity effectors, T cell independent antibodies and cytotoxic T lymphocytes (Henry et al., 2015).

## Conclusion

The impact of phages in influencing the overall wellbeing of human hosts cannot be overemphasized due to their role in impacting virulence and potential as therapeutic agents. Therefore, phages offer a promising option in the treatment of different GI diseases, particularly in conditions like CD and UC (Abraham and Cho, 2009; Nagalingam and Lynch, 2012), where phage dysbiosis has been implicated to be a developmental factor. However, further studies are required for the phage therapy to be universally accepted in clinical practice. In addition, evolving fields such as CRISPR-Cas system (Yosef et al., 2015) of bacteria provide another platform where drug delivery to host tissue can be manipulated for cancer treatment through phage-bacteria relationship. Currently, the biological function of only 25% of viral genes have been preliminarily determined (Nayfach et al., 2021). Future studies should focus on understanding the biological relevance of the human virome which can lend to better understanding of enteric disease processes and to the development of phage therapies.

## Author Contributions

All authors listed have made a substantial, direct, and intellectual contribution to the work, and approved it for publication.

## Funding

Funding provided by College of Liberal Arts and Sciences and Division of Research and Innovation Partnerships at Northern Illinois University.

## Conflict of Interest

The authors declare that the research was conducted in the absence of any commercial or financial relationships that could be construed as a potential conflict of interest.

## Publisher’s Note

All claims expressed in this article are solely those of the authors and do not necessarily represent those of their affiliated organizations, or those of the publisher, the editors and the reviewers. Any product that may be evaluated in this article, or claim that may be made by its manufacturer, is not guaranteed or endorsed by the publisher.
